# Decision making under uncertain categorization

**DOI:** 10.3389/fpsyg.2014.00991

**Published:** 2014-09-11

**Authors:** Stephanie Y. Chen, Brian H. Ross, Gregory L. Murphy

**Affiliations:** ^1^Department of Psychology, New York UniversityNew York, NY, USA; ^2^Department of Psychology, University of Illinois at Urbana-ChampaignChampaign, IL, USA

**Keywords:** categories, category-based induction, decision making, framing, uncertainty

## Abstract

Two experiments investigated how category information is used in decision making under uncertainty and whether the framing of category information influences how it is used. Subjects were presented with vignettes in which the categorization of a critical item was ambiguous and were asked to choose among a set of actions with the goal of attaining the desired outcome for the main character in the story. The normative decision making strategy was to base the decision on all possible categories; however, research on a related topic, category-based induction, has found that people often only consider a single category when making predictions when categorization is uncertain. These experiments found that subjects tend to consider multiple categories when making decisions, but do so both when it is and is not appropriate, suggesting that use of multiple categories is not driven by an understanding of whether categories are relevant to the decision. Similarly, although a framing manipulation increased the rate of multiple-category use, it did so in situations in which multiple-category use both was and was not appropriate.

## Introduction

Deciding how to act depends critically on your categorization of the situation you are in. For example, imagine pests are eating the strawberries and flowers in your garden. Should you build a fence around your garden to protect it? If a local gardener told you that your pests are very likely to be rabbits or beetles, you may have a choice of actions to take. If you categorize the pests as rabbits, building a fence seems like a good idea. The fence would keep the rabbits away and you can avoid using those harsh chemicals often used for pest control. If, instead, you categorize the pests as beetles, you would not build a fence. A fence would do nothing to protect your plants from beetles; you'll have to use those chemicals. However, given that you are not certain which of these two categories is correct, the action you should take is not clear. You could try to get further information—perhaps the bite marks look a bit large to be made by beetles—but you may end up not being certain even if you favor one pest over the other.

To make a decision when categorization is ambiguous, normatively, one should take into account all possible categorizations of the situation weighed by the likelihood that the situation belongs to that category. That is, one should integrate information across categories. To decide which action to choose, multiply the probability that the pests are rabbits by the probability that a fence would be successful in protecting your garden given that categorization. Next, multiply the probability that the pests are beetles by the probability that a fence would be successful in protecting your garden given that categorization. The sum of the two products is the probability that building a fence would successfully protect your garden from the unknown pests^1^. You would do this same thing for your other choice of action (using chemicals) and select the option with the greatest likelihood of success. This is consistent with normative principles and Bayesian approaches in which people weight different possibilities by their prior likelihoods (see Anderson, 1991, for such an approach). By considering both categories, you may arrive at a decision that is not obvious from either one alone. For example, if some chemical is not the most effective solution for either beetles or rabbits but is somewhat effective for both pests, then perhaps that solution would maximize your chance of success even if you feel that rabbits are the most likely culprits.

This paper aims to examine how people deal with such uncertainty when making decisions. Previous research in a related field, category-based induction, has found that people often only consider a single category when making *predictions* based on category information despite clearly knowing that the categorization was uncertain (Murphy and Ross, 1994; Hayes and Chen, 2008; Hayes and Newell, 2009; Murphy et al., 2012). Malt et al. (1995), Ross and Murphy (1996), and Hayes and Chen (2008) used vignettes about real-life situations to study induction when categorization is uncertain. For example, one vignette might describe an unknown person who was most likely to be a real estate agent (the *target* category) but who could have been a cable repair worker or—in a different condition—a burglar (the *secondary* category). Subjects predicted the likelihood that the unknown person would show a specific behavior, e.g., “What is the probability that the man will pay attention to the sturdiness of the doors on the house?” Since this behavior is more consistent with a burglar than a cable worker, participants receiving burglar as the secondary category should give higher probabilities than those given cable worker as the secondary category. However, in most conditions participants only paid attention to the target category (real estate agent) when making predictions, ignoring relevant information from the secondary category. Similar results have been found in studies using artificial categories.

The current project extends this category-based inference research to decision making. We had two questions. First, does decision making lead to a more rational use of multiple categories when categorization is uncertain? Second, do ideas from the decision making literature provide new ideas on how to influence thinking in these uncertain categorization situations? In particular, can we use framing effects, which often have large influences in decision making, to help overcome the reliance on single categories?

Category-based inferences are likely related to category-based decisions, as a prediction often serves as a reason for the decision. Choosing actions is perhaps one step more complex than predicting a property, because the action must be predicated upon the expected properties of the uncertain object. Thus, perhaps the results in decision making when categorizations are uncertain will show the same single-category focus as in category-based inferences. However, whereas category-based inferences ask people to make some prediction about the future, they do not point to any consequences about the decision. Because decision making involves selecting an action, people may be more careful in thinking through possible consequences. Thus, the first goal of extending the research to decision making is not simply to generalize a body of research to another related domain, but rather to ask whether this new domain, because it focuses on taking an action, might lead to a very different result.

Mynatt et al. (1993) compared two tasks that they called *inference* and *action* problems in which people had to select which evidence was most relevant to either inferring which of two categories was correct or else to deciding which of the two categories to choose. For example, they provided subjects with some evidence about a specific car and then selected more evidence in order to either say which of two kinds of car it was (inference) or which of the two cars they would buy (action). Mynatt et al. found, consistent with previous work on pseudodiagnosticity (Doherty et al., 1979; Gigerenzer and Hoffrage, 1995), that people did not collect comparable information about both categories when making an inference. But when making a decision about which car to buy in the action task, people selected information about both categories that allowed them to compare them. This result suggests that the focus on single categories found in our category-based induction task may not be found in a decision-making task. However, as we shall discuss in the General Discussion, Mynatt et al.'s design was different from ours in important ways, so the analogy is not exact.

The second goal was to use the extension to decision making to suggest additional manipulations that might promote more normative judgments, something that has proven difficult to devise with category-based inferences. In particular, we asked how framing of choice information influences how category information is used in decision making under uncertainty. Research on decision making has shown that the way choices are presented can have enormous effects on decisions, for example, loss vs. gains framing (Tversky and Kahneman, 1981, 1986) or implying one or the other choice is the status quo (Johnson and Goldstein, 2003; Stanovich, 2009, ch 7). Our goal was to attempt to discover whether use of multiple categories could be encouraged by the organization of information. If one form leads to more normative choices than another, this information could be used in designing decision frames when people must choose between uncertain options (Thaler and Sunstein, 2009).

To examine if framing of category information could promote multiple-category use, we presented information in two ways. In one way of presenting the information, the outcomes were organized by the category (the *category frame*); the possible choices and their associated outcomes were listed underneath each category. In the other organization, the information was listed by choice (the *choice frame*). In the category frame, all the choices for that category are listed together. It seems very easy to focus on the information for one category, as information about the possible categories was physically separated. In contrast, in the choice frame, this was not the case: Information about the two categories was presented together for each choice. Since choices were shared across categories, this might encourage people to combine the information across categories, as described below. Past work on category-based induction used displays analogous to the category frame; to our knowledge, nothing like the choice frame has been tested in that paradigm.

In many real-life situations, the use of categories is much more complex than in our paradigm. Fischoff and Beyth-Marom (1983) provided a classic analysis of Bayesian hypothesis testing in which they broke down the Bayes' rule into component psychological processes that were required to put it into effect (e.g., hypothesis formation, aggregating information). Our task eliminates many of these problems. For example, we specify the relevant hypotheses (categories), provide their base rates (priors), give all the information about their properties, and so on. Information search is almost trivial in our task, and memory requirements are minimal. The most relevant issue in their taxonomy is probably assessing the likelihood ratio: Comparing the evidence for one hypothesis to the evidence for another. As they point out (p. 247), the main problem in this component is that people will often attend to the evidence for a focused hypothesis and ignore the evidence for alternatives. Thus, nondiagnostic evidence may be treated as useful, if it is associated to the target hypothesis (as in Doherty et al., 1979). This is quite similar to what we have found in the category-based induction case, where evidence is derived from a target category only, even when it is not certain.

Other work has emphasized that decision making may not take into account all hypotheses that might be considered. Dougherty et al. (2010) propose a model of hypothesis generation and test in which hypotheses are first identified and then compared. Working memory provides a limit on the number of hypotheses that are considered, a number that can be reduced with task load and other manipulations. We have also considered the issue of working memory in the induction context (e.g., Ross and Murphy, 1996), and while it may affect how many categories are considered in some situations, the focus on a single category cannot be readily explained by memory limitations, as it falls well within working memory capacity. It seems likely that the limit on hypothesis generation in the real-world context that Dougherty et al. (2010) discuss is not the same as the focus on a single category in category-based induction. Indeed, the limits on the numbers of hypotheses they consider (p. 317) are 2 and 4, not 1.

Another relevant issue concerns the way in which the likelihood of the different categories is communicated. In much of our previous work, the categories were visually displayed, and subjects had to extract the base rates on their own. In the present design, which did not use perceptual features, likelihood is communicated verbally, through percentages (e.g., someone is 65% likely to have one disease and 35% likely to have another). Use of such probabilities may make it more difficult for people to reason correctly about the categories and their properties, as illustrated by research on likelihoods transmitted by probabilities, frequencies, or direct experience (e.g., Gigerenzer and Hoffrage, 1995; Hertwig et al., 2004). Furthermore, visual presentations such as those used in our past work may be especially helpful in promoting Bayesian reasoning (Arkes and Gaissmaier, 2012). Thus, it is possible that people will find it more difficult to attend to and reason about the category probabilities in the present experiment. We explicitly addressed this possibility in Experiment 2 by making sure that subjects had encoded and remembered those probabilities.

## Experiment 1

The first goal of Experiment 1 was to investigate how people use category information when making a decision under uncertain categorization. To do this, we presented subjects with short vignettes that involved a situation of ambiguous categorization. Subjects had to decide what the best choice for the main character of each story was. For example, one vignette described a situation in which a patient had to choose which medication to take for a rash with an ambiguous diagnosis (categorization), another described a professor who had to decide which book would be most useful for a student but could not remember whether the student was a business or science major. See Table 1 for an example vignette. After making a decision, subjects answered one comprehension question for each vignette. This was to ensure that subjects had carefully read the information in the story.

**Table 1:** **Sample story, Experiments 1, 2A, and 2B**.

Story text^*^
Marjorie is packing her bag for the day and remembers that she has an appointment to talk with a student from her class about career options. She wants to bring her student a book that is relevant to her interests. The problem is that Marjorie only has room in her bag for one book and she can't remember which of her students she is speaking with today, because her calendar was erased in a computer malfunction. Her class is made up of 65% (95%) science majors and 35% (5%) business majors. The types of jobs they are looking for are very different. The two books (one by Jones, the other by Smith) about careers for recent college graduates that Marjorie has focus on different topics and are differentially useful for the different majors.^**^
Please press the next button to answer a question before you see this information and help Marjorie make a decision about what book to bring.
**Category frame**
**SCIENCE MAJORS (MORE LIKELY)**
Jones book is useful for 41% of science majors
Smith book is useful for 65% of science majors
**BUSINESS MAJORS (LESS LIKELY)**
Jones book is useful for 78% of business majors
Smith book is useful for 3% of business majors
**Choice frame**
**USEFULNESS OF SMITH BOOK**
Smith book is useful for 65% of science majors (more likely major)
Smith book is useful for 3% of business majors (less likely major)
**USEFULNESS OF JONES BOOK**
Jones book is useful for 41% of science majors (more likely major)
Jones book is useful for 78% of business majors (less likely major)
Decision question^***^
If you were Marjorie, which book would you take?
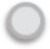 Jones 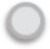 Smith
Reading comprehension question^***^
What is Marjorie's profession?
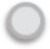 lawyer 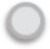 editor 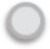 professor 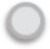 librarian

* *Percentages outside of parentheses were used in the high uncertainty condition. Percentages in parentheses were used in the low uncertainty condition*.

** In Experiments 2A and 2B this sentence was changed to, “The three books (one by Jones, one by Kendall, and one by Smith) about careers for recent college graduates that Marjorie has focus on different topics and are differentially useful for the different majors.”

*** *Order of answer options randomized for each subject*.

Following the design of Murphy and Ross (2010b), we constructed the stories such that we could determine whether subjects were basing decisions on multiple categories or on only a single category. One choice was dominant if subjects considered only the most likely or *target* category, and another choice would be the dominant choice if subjects also considered the less likely or *alternative* category in addition to the target category. In the story in Table 1, the target category (science majors) was 65% likely, and the alternative category (business majors) was 35% likely. If one only paid attention to the target category, one would decide to bring the Smith book, because it was useful for more science majors (65% of them) than the Jones book (41%). However, if paying attention to both categories, the best option was the Jones book. The probability that the Jones book would be useful was the probability of the student being a science major × the proportion of science majors that find the Jones book useful plus the same product for business majors (65% × 41% + 35% × 78% = 54%). The same calculation for the Smith book yields a lower outcome (65% × 65% + 35% × 3% = 43%), thus, bringing the Jones book was the better choice if both possible categorizations of the situation were taken into account.

These calculations follow the general Bayesian rule that the prediction of each category should be weighted by its likelihood (e.g., Anderson, 1991). However, the predictions do not depend on such weighting, which could be computationally difficult. For example, if people give equal weights to the categories, then they should also choose the Jones book, because it has moderately high success rates in both categories (i.e., the average of 41 and 78% for Jones is greater than the average of 65 and 3% for Smith). These types of simple averaging strategies will, of course, not always be successful (see McKenzie, 1994 for analysis of the performance of various averaging strategies vs. Bayesian ones). For example, if people arbitrarily give weights that differ greatly from the stated probabilities, the predictions no longer hold. However, in past research in which we have asked subjects to provide their own probabilities for the categories, they are generally quite reasonable (e.g., Murphy and Ross, 2010b).

The second goal of Experiment 1 was to examine how the framing of choice information influences subjects' propensity to use multiple categories in decision making. To examine this issue, we framed the provided information in two ways, by category or by choice. The above calculations do not depend on how the various probabilities are presented. As Table 1 suggests, the category frame might encourage subjects to focus on the most likely category and therefore not even notice that one of its associated choices is not useful to the alternative category. In contrast, the choice frame presents information about a given choice together, putting information about the usefulness of that choice for all possible categories together, thereby possibly encouraging people to use multiple categories. In order to only pay attention to one category in this frame, subjects would have to selectively ignore a given category's information separately for each header.

Thus far we have described what we will call the *high uncertainty condition*. As in the example above, the multiple-category choice is the best response in all decisions made in that condition because there is considerable uncertainty surrounding the categorization of the situations in these stories. However, selection of the multiple-category choice does not necessarily mean that the subject carefully weighed the two categories or realizes that this choice maximizes the chance of success—it could have arisen through random choice or a suboptimal single category strategy (see Murphy et al., 2012, Experiment 2). Thus, it is important to understand if a manipulation that increases multiple-category use does so in the appropriate situations. To examine this we included an additional condition not used in previous studies, the *low uncertainty condition*, in which only the information in the target category should be considered. In the low uncertainty condition, there was almost no uncertainty about what category the situation belonged to. The target object was 90–95% likely to be in the target category, so subjects should select the dominant choice for the target category (e.g., the Smith book)^2^.

There are many ways to test decisions when categories are uncertain. As described above, one technique is to use familiar categories (e.g., real estate agent and burglar) and require subjects to retrieve the necessary information from semantic memory (e.g., how likely real estate agents are to examine the doors on a house). Such methods have generally found very little evidence for use of multiple categories (e.g., Ross and Murphy, 1996). Providing a visual display of categories has found that about 20–45% of subjects use multiple categories, depending on the materials and subject population (Hayes and Newell, 2009; Murphy and Ross, 2010b). The present technique involves presenting subjects with a table-like format of probabilities, which controls for any differences in subjects' knowledge and removes any reliance on memory. Certainly, providing explicit probabilities and outcomes is very common in decision-making tasks (e.g., gambles). However, there is a chance that the mere presentation of these probabilities will encourage people to use multiple categories, as found in an experiment on induction that used a similar format (Murphy and Ross, 1999). The low uncertainty condition will help to put such a result, should it occur, into perspective. That is, it will help to reveal whether use of multiple categories is thoughtful or instead a response to presentation format that occurs even when it is not appropriate.

### Method

#### Subjects

Subjects in all experiments were recruited via Amazon Mechanical Turk. Each Mechanical Turk worker was allowed to do each study only once (workers were identified by their worker ID). Worker IDs were recorded for each study so that we could prevent workers from participating in more than one of the studies reported in this paper. All subjects gave informed consent, and the study was approved by the New York University Institutional Review Board.

The experiment lasted approximately 10–15 min and subjects were paid for their participation. Seventy-two subjects were randomly assigned to conditions in Experiment 1. Subjects had to answer four out of the six reading comprehension questions correctly to be included in the analysis. All subjects in Experiment 1 reached this criterion.

#### Materials and design

The design included two between-subject factors: frame (category frame, choice frame) and category order (target category information presented first or second). The design also included one within-subjects factor: category uncertainty (high uncertainty, low uncertainty).

There were six short vignettes. A sample of one of these vignettes is provided in Table 1 (see Supplementary Materials online for all vignettes). The stories described a scenario in which the main character had to make a decision about what choice would be best given his or her situation. However, the categorization of the character's situation was ambiguous. For the story in Table 1, a meeting could be either with a student who studies science or with a student who studies business. The only difference in the story text between the high uncertainty and low uncertainty conditions was that the situation was 60–65% likely to be in the target category in the high uncertainty condition and 90–95% likely to be in the target in the low uncertainty condition. (We varied the probabilities slightly within each condition so that subjects would not be repeatedly answering the exact same problem.)

The stories included information about two possible choices that the main character could take. The choices were differentially effective for the two possible categorizations of the situation. One choice was the dominant option for situations belonging to the target category (for the story in Table 1, bringing the Smith book for science majors) and the other was the dominant choice for situations belonging to the alternative category (bringing the Jones book for business majors). The choice that was dominant for the alternative category was the overall best choice if basing the decision on multiple categories, thus we assumed that selection of such a choice indicated use of multiple categories (we explicitly confirm this in the second experiment).

There were two versions of how the choice information was presented for each story. In the category frame, information about each action was grouped by category (e.g., each of the majors). That is, there was a header for each category and underneath it the effectiveness or usefulness of each choice for that category was listed. In the choice frame, information about each category was grouped by choice. That is, the headers were now the names of the choices (which book to bring) and information about the choice's effectiveness or usefulness for each category was listed under this header (see Table 1 for examples). Each frame explicitly indicated which category was most likely so that this information would be equally salient. For each frame, there were two orders that the category information could be presented in: Target category information could either be presented first (on top), or second (on bottom). The target category information is presented first in the example shown in Table 1.

#### Procedure

Instructions stated that subjects would read six short stories and make decisions about what choice the main character of each story should make. Each story would be accompanied by information about the possible choices that should be used in making their decision.

Order of vignettes was randomized for each subject. Each subject read four high uncertainty vignettes and two low uncertainty vignettes. The vignettes that were assigned to each certainty condition were counterbalanced across subjects. Story text was presented on its own screen. Once subjects read the story, they clicked on the “next” button with their mouse to see that information about the possible choices (story text was also presented on this screen). When they pressed the “next” button, a question appeared at the bottom of the same screen that asked what choice they would make if they were the main character in the story. The two choices were presented below the question (order of answer options was randomized on each trial). Subjects clicked on the radio button next to one choice to answer.

For each story, one reading comprehension question was asked after subjects made their decision. This multiple-choice question was presented on a separate screen and asked about a fact included in the story or an inference that could have easily been made had the subject read the story. These questions were intended to encourage close reading of the stories. See Table 1 for an example. Trials in which subjects answered incorrectly were excluded from analysis.

### Results

The dependent measure was the proportion of multiple-category choices made in each condition. The results were subjected to a 2 × 2 × 2 (Frame × Category order × Category uncertainty) ANOVA. There was no evidence that framing influenced predictions. The main effect of frame was not significant, *F*_(1, 68)_ = 1.3, *p* > 0.05. Category order entered into no significant effects, so all results will be presented collapsing over the target category information presented first and second.

The results revealed that people took into account the category likelihood when making decisions. They selected the multiple-category response far more in the high uncertainty condition than in the low uncertainty condition (*M*s = 55.9 and 24.3%), *F*_(1, 68)_ = 38.6, *p* < 0.01. Note that these means show that subjects often did not integrate information across category when making decisions under uncertainty. In the high uncertainty condition, subjects chose the multiple-category choices only slightly more than half of the time. In the low uncertainty condition, when subjects should only be paying attention to the target category, subjects also selected the multiple-category option a surprising amount, almost one quarter of the time.

**Figure 1:**
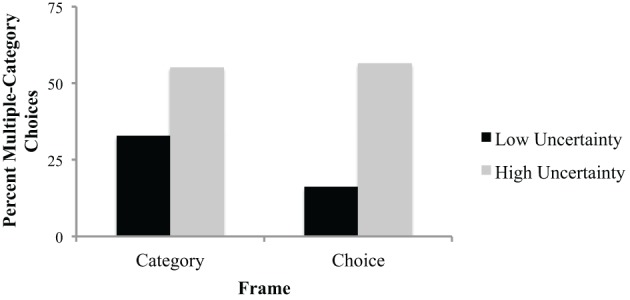
**Mean percent of multiple-category choices as a function of frame and category uncertainty, Experiment 1**.

The Category uncertainty × Frame interaction was marginally significant, *F*_(1, 68)_ = 3.0, *p* = 0.087. The means revealed that subjects in the choice frame condition were more sensitive to the certainty manipulation than those in the category frame condition. That is, those in the choice frame condition showed a greater difference in the percent of multiple-category choices between the high and low uncertainty conditions than those in the category frame (see Table 2 and Figure 1).

**Table 2:** **Mean percent of multiple-category choices, Experiment 1**.

**Category uncertainty**	**Category frame**	**Choice frame**	**Mean**
Low uncertainty	32.9	16.2	24.3
High uncertainty	55.2	56.5	55.9
Mean	47.7	43.6	

#### Best choice analysis

The above analysis used the percent of multiple-category selections as the dependent measure. However, the multiple-category choice was the best choice only in the high uncertainty condition. In the low uncertainty condition the target category was so likely that the best choice for the target category was the best choice overall (using a Bayesian calculation of the likelihood of success for each action—see footnote 2). It is of interest to ask which condition led to normative performance, under the assumptions just described. A 2 × 2 × 2 (Frame × Category uncertainty × Category order) ANOVA was performed with the percentage of best choice selections as the dependent measure. No interaction was significant, so we will focus on the main effects.

Subjects were 19.8% more likely to select the best choice in the low uncertainty than the high uncertainty condition, *F*_(1, 68)_ = 8.6, *p* < 0.01. The main effect of frame was marginally significant, *F*_(1, 68)_ = 3.0, *p* < 0.087, as subjects were more likely to select the best choice in the choice frame. See Table 3 for details. Order of category presentation did not influence how likely subjects were to make the best choice, *F*_(1, 68)_ = 0.4.

**Table 3:** **Mean percent of best choices, Experiment 1**.

**Category uncertainty**	**Category frame**	**Choice frame**	**Mean**
Low uncertainty	67.1	83.8	75.7
High uncertainty	55.2	56.5	55.9
Mean	61.2	70.2	

### Discussion

The results of Experiment 1 revealed that subjects were not particularly good at identifying when multiple categories were and were not relevant in making decisions. In the high uncertainty condition, they only picked the multiple-category choice approximately half of time when it was the best choice. In the low uncertainty condition, where they should have only considered the target category, subjects selected the multiple-category choice almost one-quarter of the time. It seems odd that they would be integrating information across categories when they did not need to, given how often they did not integrate when they should have. However, it is possible that the selection of the multiple-category choice in the low uncertainty condition was not a result of true consideration of multiple categories. Perhaps subjects were not attending to the category base rates carefully. Some of these multiple-category choices could have been the result of either random choice, or mistakenly only paying attention to the alternative category and simply picking the dominant choice for that category. Since the dominant choice for the alternative category was the same as the multiple-category choice there is no way to differentiate between these two possibilities. Experiments 2A and 2B address this issue.

As remarked earlier, it may be that the technique of presenting probabilities for all the outcomes in tabular form causes some people to do some sort of averaging over all the categories whether or not all of them should be considered. For example, if the Smith book is useful 65 and 3% across science and business majors, respectively, it doesn't seem as useful (34% on average) as the Jones book that is useful 41 and 78% of the time (60% useful on average). But simple averaging is not always called for. If the student is very unlikely to a business major, as in our low uncertainty condition, then Smith's book is more likely to be useful than Jones's (65 vs. 41% for science majors). Therefore, when subjects chose the multiple-category choice even when uncertainty was low, they may have been engaging in unwarranted averaging. This suggests that some of the 55.9% of multiple-category use in the high uncertainty condition may have arisen from the same process. We discuss this possibility in the General Discussion.

There was a hint that how the information was framed influenced how likely subjects were to pick the best choice: Subjects were 9% more likely to select the best choice in the choice frame, but this difference did not reach statistical significance. Clearly, the effect is not very large, if it does exist. Additionally, subjects were more likely to select the best choice in the low uncertainty condition than in the high uncertainty condition. This is perhaps not surprising as selecting the best option in the low uncertainty condition does not involve integration of information across categories and only involves consideration of the target category information. Thus, this finding is similar to past results from the category-based induction literature.

## Experiments 2A and 2B

Subjects may engage in a kind of probability matching in which they choose the less likely category as the correct one in a minority of trials. In Experiment 1, the most likely choice in the less likely category was also the one that would be selected if subjects attended to multiple categories. To allow us to differentiate true multiple-category choices from choices that were the result of a focus on the alternative category, we added a third choice to Experiments 2A and 2B. In these experiments there was one choice that was dominant for the target category, one that was dominant for the alternative category, and one that was best if attending to both categories (analogous to a design used with visually presented categories in Murphy et al., 2012, Experiment 4). To further ensure that subjects' multiple-category choices were not the result of ignoring the category base rates, we required subjects to correctly report them prior to making their decision. This ensured that subjects had read and taken note of the base rates.

Experiments 2A and 2B differed in only two ways. First, Experiment 2B included a question about which category subjects thought the ambiguous situation most likely belonged to prior to the base rate question. Second, the base rate question in 2B only asked about the most likely category. The categorization question aimed to further ensure that subjects understood the relative base rates of the target and alternative categories. Additionally, this question could provide some insight into the effect of categorization on the use of multiple categories in decision making. As literature on category-based induction has suggested that categorizing an ambiguous item prior to making an induction may promote single-category reasoning (Hayes and Newell, 2009; Murphy et al., 2012), it will be important to understand its effect on decision making. The two experiments were run separately, but because they used the same critical questions and had similar results, we present them together.

### Method

#### Subjects

Seventy-seven subjects were randomly assigned to conditions in Experiment 2A; data from one subject was dropped for answering more than two reading comprehension questions incorrectly. Ninety-nine subjects were randomly assigned to conditions in Experiment 2B; data from three subjects were dropped for answering more than two reading comprehension questions incorrectly.

#### Materials and design

The six vignettes from Experiment 1 were used. The story text was identical to that in Experiment 1 (as shown in Table 1) except that the stories now included information about three possible choices that the main character could make (see Table 4). One choice was dominant for situations belonging to the target category (for the story in Table 1, bringing the Smith book for science majors), one choice was dominant for situations belonging to the alternative category (bringing the Kendall book for business majors), and one choice was not dominant for either category. In the high uncertainty condition, this choice (bringing the Jones book) was the best choice overall because of the uncertainty of categorization.

**Table 4:** **Sample choice information, Experiments 2A and 2B**.

**Category frame**
**SCIENCE MAJORS (MORE LIKELY)**
87% Find Smith book useful
1% Find Kendall book useful
66% Find Jones book useful
**BUSINESS MAJORS (LESS LIKELY)**
3% Find Smith book useful
96% Find Kendall book useful
86% Find Jones book useful
**Choice frame**
**USEFULNESS OF SMITH BOOK**
Useful for 87% of science majors (more likely major)
Useful for 3% of business majors (less likely major)
**USEFULNESS OF KENDALL BOOK**
Useful for 1% of science majors (more likely major)
Useful for 96% of business majors (less likely major)
**USEFULNESS OF JONES BOOK**
Useful for 66% of science majors (more likely major)
Useful for 86% of business majors (less likely major)

As in Experiment 1, these choices were presented in two ways, grouped by category or by choice, as shown in Table 4, and there were two orders that the category information could be presented, target category information could either be presented first (on top), or second (on bottom). As in Experiment 1, the correct category had either high or low uncertainty. These three factors were between-subjects manipulations.

The choices appeared in three orders: target's dominant choice first, alternative's dominant choice first, or the multiple-category choice first. We created three sets of stories such that each set contained two stories from each of the possible choice orders. Each subject was assigned one of these three sets of stories.

#### Procedure

The procedure was identical to that used in Experiment 1 except for the addition of the base rate question and the most likely category question (Experiment 2B only). In Experiment 2A, once subjects were done reading the story, they were asked (on a separate screen) what the base rate of each category was. They were told that the base rates of the two categories had to add to 100%. If they answered incorrectly, they were told to re-read the story and try again. Trials in which subjects answered incorrectly more than three times were excluded from analysis. Once they answered the base rate question correctly, subjects read information about the possible choices and the effectiveness or usefulness of that choice for each category as shown in Table 4 (story text was also presented on this screen), and the experiment proceeded as it did in Experiment 1.

In Experiment 2B, after reading each story, on a separate screen subjects were asked which category the situation most likely belonged to. Once they got this question correct they then reported (on a separate screen) the base rate of the most likely category. If subjects got the base rate question incorrect, they returned to the story and had to answer both the categorization and base rate question again. Any trials in which subjects took more than three attempts to get both questions correct were dropped.

### Results

Performance on the base rate and categorization questions was high. In Experiment 2A, subjects took, on average, 1.1 attempts to get the base rate question correct. Subjects answered 90.8% of trials correctly on their first attempt. In Experiment 2B, the average number of attempts was also 1.1, and subjects answered 92.2% of trials correctly on their first attempt.

The results of Experiments 2A and 2B were analyzed together with experiment (2A vs. 2B) as a between-subjects factor. A 2 × 2 × 2 × 2 (Frame × Category uncertainty × Category order × Experiment) ANOVA was performed with the percentage of multiple-category choices as the dependent measure. No interaction was significant, so we focus on the main effects.

As predicted, subjects chose the multiple category action more in the high uncertainty (*M* = 70.4%) than the low uncertainty condition (*M* = 33.5%), *F*_(1, 156)_ = 49.7, *p* < 0.01. As with Experiment 1, subjects selected the multiple-category choice a significant proportion of the time in the low uncertainty condition when the dominant choice for the target category was the best option. It should also be noted that subjects rarely picked the dominant choice for the alternative category (only 1.7% of the time).

The main effect of frame was also highly significant, *F*_(1, 156)_ = 8.0, *p* < 0.01. Subjects chose the multiple-category choice more in the category frame (*M* = 59.2%) than in the choice frame (*M* = 44.5%). The main effects of experiment and category order were not significant, suggesting that the addition of the most likely category question and the order in which category information was presented did not affect subjects' propensity to use multiple categories in their decision making (see Table 5 for details). Although the means in Table 5 may suggest that the effect of uncertainty differed across frames, this interaction was not significant, *F*_(1, 156)_ = 1.2.

**Table 5:** **Mean percent of multiple-category choices, Experiments 2A and 2B**.

	**Category uncertainty**	**Category frame**	**Choice frame**	**Mean**
Experiment 2A	Low uncertainty	46.3	24.2	35.2
	High uncertainty	83.9	72.3	78.1
Experiment 2B	Low uncertainty	42.0	24.0	33.0
	High uncertainty	67.9	61.3	64.6
Mean		59.2	44.9	

#### Individual response patterns

We categorized subjects into three different groups based on their response strategy, as shown in Table 6. Subjects were either multiple-category responders (selected the multiple-category choice for all or all but one trial), single-category responders (selected the target category choice for all or all but one trial), or were mixed in their strategy (all other patterns of choices). Almost three-quarters of subjects were consistent in their responses (i.e., were either single- or multiple-category responders). A chi-square test revealed that the distribution of strategies was significantly different between the high and low uncertainty conditions, such that more subjects in the high uncertainty condition were multiple-category responders, χ^2^(2, *N* = 172) = 41.9, *p* < 0.01. The choice patterns did not differ significantly for the category vs. choice frame, χ^2^(2, *N* = 172) = 4.4, *p* > 0.05, though the difference was in the direction of more multiple-category responders in the category frame, as in the percentage analysis above.

**Table 6:** **Distribution of response strategies, Experiments 2A and 2B**.

	**Multiple-category responders**	**Mixed responders**	**Single-category responders**
Low uncertainty	14	30	43
High uncertainty	54	17	14
Total	68	47	57
Category frame	39	24	22
Choice frame	29	23	35
Total	68	47	57

#### Best choice analysis

**Figure 2:**
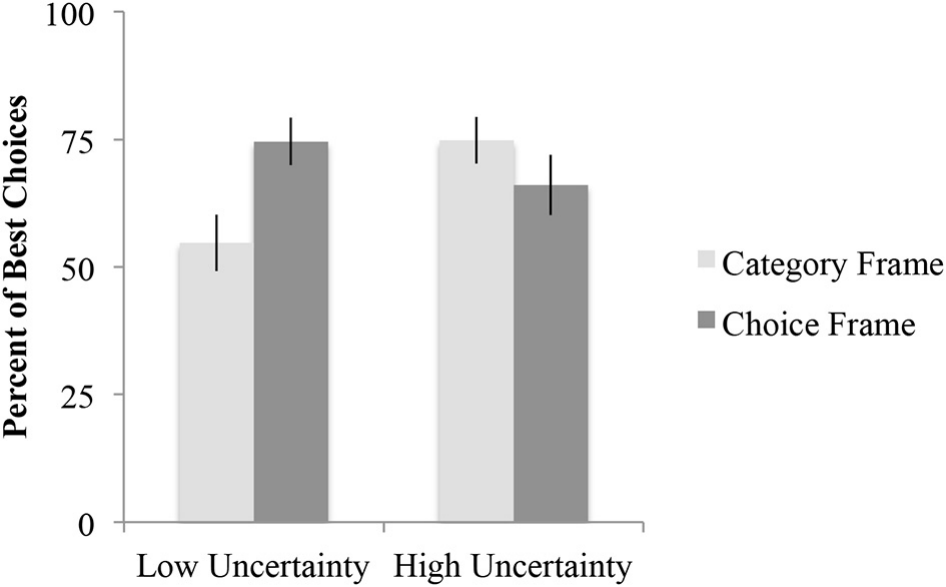
**Mean percent of best choices for each frame and category uncertainty**. Error bars represent the standard error of the mean.

A 2 × 2 × 2 × 2 (Frame × Category uncertainty × Category order × Experiment) ANOVA was performed with percentage of best choice selections (defined as in Experiment 1) as the dependent measure. The only significant result was the Category uncertainty × Frame interaction, *F*_(1, 156)_ = 7.7, *p* < 0.01. As can be seen in Figure 2, this interaction was the result of the category frame slightly increasing (by 9%) the frequency of best choice selections relative to the choice frame in the high uncertainty condition but decreasing it (by 20%) in the low uncertainty condition. Tests of the simple main effects of frame revealed that the difference in percent of best choice selections between the category frame and choice frame was significantly different in the low uncertainty condition, *t*_(85)_ = −2.8, *p* < 0.01, but not in the high uncertainty condition, *t*_(83)_ = 1.1, *p* > 0.05.

### Discussion

Under high uncertainty, the category frame promoted use of multiple categories in decision making. However, the category frame increased *overall* multiple-category use, both when it was and was not appropriate. Thus, although the category frame promoted multiple-category use, it did so at the expense of accuracy in a situation in which only a single category is relevant to the decision. This pattern of results is surprising. Intuitively, it seems that presenting the information about different categories together should encourage use of multiple categories, whereas both experiments found greater multiple-category use when the categories' information was separated.

The categorization of the situation prior to making the decision did not significantly impact the amount of multiple-category use. This is a bit surprising given that this has been found to promote single category reasoning in category-based induction (Hayes and Newell, 2009; Murphy et al., 2012; Chen et al., 2014). In these past studies subjects were generally not required to answer these questions from memory, nor were they required to answer these questions correctly in order to move on in the experiment. The procedure used in Experiment 2B may have weakened the influence of the categorization question by quizzing the subject on the base rate of the target category, and perhaps also by asking them a comprehension question. Subjects were never told that we would only ask for the base rate of the target category after each vignette (i.e., they may have thought that there was some chance that we may ask about the alternative category at some point) and the topic of the comprehension question varied quite a bit. This may have caused subjects to pay more attention to the story overall and the base rates of both categories in order to correctly answer the questions, thus weakening the effect of the categorization question.

Recall that in Experiment 1, the multiple-category choice was also the best one for the alternative category, creating some ambiguity as to whether people were actually using multiple categories. The use of three options in Experiment 2 clarified these results, as Experiment 2 subjects very rarely chose the option that was best only for the alternative category. Rather, they often selected the choice that was good in multiple categories, even when one of those categories was very unlikely. Thus, the multiple-category choices in Experiment 1 probably reflected true use of multiple categories.

The proportion of multiple-category responses seemed to increase from Experiment 1 (40% overall there, compared to 52% here). It may be that the addition of a new option with two new properties caused some subjects to think that multiple pieces of information must be relevant. That is, now that there were six percentages presented, rather than four, the tendency to focus on only one of them may have been reduced, under the assumption that more than one of the six pieces of information must be relevant to the answer.

Finally, subjects were overall quite consistent in their responding, with 73% of them responding consistently. This cannot be readily compared to the rates of previous work (Murphy and Ross, 2010b), which had different numbers of items and correspondingly different rules for consistency. The present design was completely between-subjects, which no doubt encourages people to form a strategy and stick to it. It is possible that if people had a variety of problem types to respond to, their answers would have been more diverse.

## General discussion

The goals in this present research were to examine whether decision making showed the same suboptimal single-category focus often found in category-based induction tasks and whether framing could influence use of category information. We found that although subjects tended to choose the multiple-category choice when it was the best option (in the high uncertainty condition), they often selected this option when it was not the best option as well (in the low uncertainty condition). Framing influenced how category information was used, such that a category frame led to greater use of multiple categories (Experiments 2A and 2B). However, importantly, our results also revealed that this increase in multiple-category use was indiscriminate. Overall the results suggest that multiple-category use is often not a result of a deeper understanding of when information is and is not relevant.

### Category-based induction vs. category-based decision making

This work was inspired by previous research on category-based induction under uncertainty that has consistently found suboptimal use of category information (Murphy and Ross, 1994, 2010b; Ross and Murphy, 1996). The main uncertainty in both category-based induction tasks and the present task involved the categorization of a critical object or person. Such uncertainty is certainly a common event in everyday life, as when one doesn't know which event will occur or which object will appear. Nonetheless, actions often have to be taken in the absence of that knowledge, and taking into account multiple possibilities is often the best way to maximize performance (Anderson, 1991; but see Huys et al., 2012 for a discussion of the utility of focusing on fewer possibilities in decision making).

We extended our work on category-based induction under uncertainty to a parallel decision-making task. However, such extensions also involved a number of changes to our usual procedures. Subjects chose among actions that would be more or less successful, depending on the correct category. In past work on category-based induction, the choice was among properties of the category. Referring to our original example of the unknown pest that is eating up your garden, a category-based induction task might ask about a property of the unknown pest (e.g., about its size), rather than if you would build a fence or spray chemicals. Of course, these two tasks are related, as the decisions are likely predicated on the inferences you make (e.g., you choose to build the fence because you have inferred that it will successfully protect your garden based on the properties of the pest).

Another major change from the traditional category-based induction tasks is that we explicitly provided probabilities of the uncertain categories as well as the probability of “success” of each choice given the category rather than requiring subjects to infer these probabilities from displays or from memory. This way of presenting information is similar to many decision-making tasks that involve gambles or other uncertain choices in which probabilities and outcomes are explicitly listed. We speculate that this change in format may promote multiple-category use compared to the formats generally used in category-based induction tasks and may account for some of the high rate of multiple-category use discussed in the next section.

#### Multiple-category use in decision making

Our first goal was to examine whether decision making under uncertain categorization avoids the suboptimal single-category focus often found in category-based induction research. Although differences in the paradigms used prevent direct comparison of their results, our results revealed that subjects in our experiments did not tend to show the suboptimal single-category focus. The use of multiple categories was quite high in the high uncertainty condition, especially in Experiments 2A and 2B. This high level of performance cannot be interpreted uncritically as indicating accuracy, however, because of the results of the low uncertainty condition.

When one knows the category of a critical object, one should clearly not use information about other categories in making a decision. For example, if one is sure that an object is a horse, then information about cows should not influence what action one takes toward the object^3^. In our low uncertainty condition, we did not use absolute certainty but probabilities that the object was in the target category of 90% and above. Either a general heuristic of ignoring low probabilities or an actual calculation of the best option using the probabilities would indicate that the choice dominant in the target category would be best. Subjects who chose the “multiple-category” choice (here somewhat misnamed because the best choice when paying attention to multiple categories was actually the target category's dominant choice) must therefore have been engaging in a strategy in which they simply averaged together two probabilities whether or not they both should have been considered.

There seem to be two possible reasons for such a strategy: failure to realize that an alternative is not relevant or failure to ignore an unlikely alternative despite noting that it is irrelevant. Failure to notice the category base rates could result in a failure to realize that the alternative is not relevant. If one assumed that both categories were fairly likely, then considering both would be sensible. This is why we introduced the check of base rates in Experiment 2. On every trial, subjects had to indicate the probability of both categories (Experiment 2A) or of the target category (Experiment 2B) so that failure to encode these likelihoods was no longer a possibility. On a significant number of trials, people indicated that the target category was (say) 92% likely to be correct, and the alternative 8% likely, and then went on to give significant weight to the alternative option, overriding the choice that would be best for the target category. Thus, it seems that the over-use of information from unlikely outcomes arises, at least in part, from a failure to ignore these alternatives despite knowing (and being required to state) that they are very unlikely.

The use of the alternative categories in these decision indicates an unthinking tendency to pay attention to the information whether or not it is truly relevant: If the display provides information for a category, then that information will be used, even if the category is very likely not correct. We suspect that this tendency occurs much more with displays like ours that present all the probabilities than in cases where people must retrieve information from memory or calculate it from a display (as in our past work, where multiple-category use was much less; Murphy and Ross, 2010b; Murphy et al., 2012). Such displays might have similarly undesirable effects in real-life examples as well. For example, if an article on investments presents information for different kinds of investors or for different market conditions, people might well attend to all the information whether or not it is relevant. They might conclude “sometimes stocks are better and sometimes bonds are” even if it's indicated that one or the other is clearly best for their own situation or for the economy expected in the next few years. In medical decisions, one might be influenced by outcome statistics on conditions that are unlikely to be the correct diagnosis (categorization) given the patient's symptoms. We discuss how this research may inform such real-world situations in the Recommendations section.

The over-use of information from unlikely outcomes has led us to re-think how people use multiple categories in situations where it is appropriate to do so. In asking whether people use multiple categories, the assumption has been that they do so because of some understanding that integrating predictions across categories (ideally, weighted by their probability) leads to better choices than using only a single category. But this assumption seems dubious if people do the same thing when it leads to worse choices. Similarly, Beyth-Marom and Fischoff (1983, Experiment 4) found that most people who correctly identified information about an alternative hypothesis as being relevant then provided an incorrect reason for its relevance.

Given that the tendency to use multiple categories in the low uncertainty condition is an estimate of the unthinking use of multiple categories (the *averaging strategy*), we can estimate the proportion of responses in the high uncertainty condition that are due to some understanding of the principle by subtracting this “error” from those conditions. For example, in Experiment 1, 56% of subjects selected the multiple-category option in the high uncertainty condition, but some of these choices were very likely due to this averaging strategy. Given that subjects used that strategy 24% of the time in the low uncertainty condition, when it wasn't appropriate, this suggests that only 32% (56–24%) of the multiple-category responses were based on appropriately weighting the two categories in the high uncertainty condition. The same subtraction method yields an estimate of 37% in Experiment 2. Thus, for this paradigm, a reasonable estimate of how often people are integrating across categories (rather than simply averaging any numbers that are displayed) is 25–40%. This is similar to estimates of effects in category-based induction, which also vary across paradigms and between labs, depending on details of the problems and subject populations (Hayes and Chen, 2008; Hayes and Newell, 2009; Murphy and Ross, 2010b). Thus, although people tend to pay more attention to the alternative category than they generally do in category-based induction tasks, the rate of multiple-category use once these unthinking instances of it are excluded does not seem very different in these two tasks.

This effect can be related to well-known examples of base-rate neglect (e.g., Kahneman and Tversky, 1973; Tversky and Kahneman, 1980). We went to some effort in Experiment 2 to make sure that subjects encoded the frequency differences between the categories by asking them to provide the relevant category frequencies. One might well think that those questions would serve to alert subjects to the fact that base rates were important. However, this did not stop them from using the information from categories that they had correctly identified as being unlikely a third of the time. This situation contrasts with cases in which base rates are only implicit in the question (e.g., Kahneman and Tversky's question about college majors), requiring subjects to spontaneously think about the relevance of base rates and then retrieve them. We provided the information and tested subjects on it. More likely, this finding is a result of the salience of the categorical information. When trying to choose a book, some subjects examined all the information about each book that was on the screen, regardless of its relevance. The base rate had been tested moments ago and was no longer present—the averages for each option were visually present. Of course, not every person did this, but the effect is reminiscent of other forms of “mindlessness” we have discovered in the induction task, such as using cues that were known to be random (Murphy and Ross, 2010a) or focusing on categories mentioned in a question, even though the question provided no information about the induction (Murphy et al., 2012). Those effects were also found in a minority of subjects, but they all reflect the influence of salient information that was not actually relevant to the task at hand.

In the Introduction, we discussed an important experiment by Mynatt et al. (1993) that directly compared induction and decision making in an information-selection task. They found (Experiment 1) a large difference between these two tasks on the dependent measure of whether people adequately tested multiple hypotheses. Their result is generally consistent with our finding that people seem to use multiple categories quite often in the present decision-making task (e.g., 60–80% of the time in Experiment 2, high uncertainty condition, which is most analogous to their situation), in contrast to earlier work on induction. However, their task was importantly different from ours in a number of respects. First, the dependent measure was information sampling, not making a prediction or choice. We provided subjects with all the available information, whereas they required subjects to choose which information they wished to receive for each category. Second, the action required in their decision-making condition was essentially a choice of one of the two categories. For example, subjects were told about two kinds of cars and were asked to imagine that they were going to choose which car to buy. In our task, the choice was not between the categories but rather about actions that were associated to various degrees with the categories. One could choose an action by looking at only one category. Their task explicitly required people to examine both categories in order to select one, which was exactly the motivation for their prediction that people would consider multiple hypotheses in the decision-making task.

In short, although our results are consistent with Mynatt et al.'s, the dependent measures and information provided were so different that the effects are by no means identical. Furthermore, in Mynatt et al.'s design, sampling multiple categories was always a normatively correct answer. In our design, it does not appear normative to combine information across categories in the low uncertainty condition, and yet people often did so, as just discussed. Thus, our results provide a new finding that could not be discovered in Mynatt et al.'s paradigm.

As we remarked in the Introduction, our task simplifies many of the aspects of decision making that can cause difficulties. One important issue is that we provided all the categories, in part as a matter of experimental control. However, in related hypothesis-testing research, generation of the alternatives can be a significant problem. For example, when trying to solve open-ended problems, people are not very good at providing possible actions. Gettys et al. (1987) found that individual undergraduate subjects could only generate about half of the useful actions to help address a realistic problem. Dougherty et al. (2010) argue that working memory limitations reduce the number of hypotheses considered as well as increase the tendency to focus on the current hypothesis in probability estimation. Obviously, one's ability to integrate information across alternatives is greatly reduced if the alternatives are never considered to begin with. On the other hand, when the alternatives involve categorizing a person or situation, it may be that semantic memory is sufficient to provide access to the relevant categories. Dougherty et al.'s HyGene model provides insight into that process. More research on open-ended induction and decision-making problems are needed to identify to what degree category generation is adequate in such situations.

A final connection to decision-making research is Shafir's (1994) analysis of disjunctive situations. In a number of studies, he has found that people find it difficult to choose an action when they don't know which of two situations will occur—even when the preferred action is identical in those situations. In one well-known example, students expressed a desire to (hypothetically) buy a vacation package when they knew the outcome of a major exam, both when they knew they had passed and when they knew they had failed. But when told that they didn't know whether they had passed or failed the exam, many did not choose the vacation package (Tversky and Shafir, 1992). It seems that people do not understand that they can come to an answer in such situations by considering all the options and seeing if there is a predominant choice. Toplak and Stanovich (2002) report even greater problems for some analogous disjunctive reasoning tasks. This appears to conflict with our finding that some subjects did combine information across the two potential categories even when it was not wise to do so. Again, we suspect that the visual presentation of the options is probably responsible for this difference. If Tversky and Shafir's stimuli had spelled out the disjunctions, the problems would have become trivial (e.g., if you pass the exam, you would take the vacation; if you fail, you would take the vacation; if you don't know whether you have passed or failed, would you take the vacation?). Our problems were not trivial, because the best choice was different in the two categories, but we think it is likely that presenting the options explicitly has a strong effect on what information people attend to.

#### Framing effects

The other main goal of this work was to examine whether different ways of presenting the same information would encourage use of multiple categories. We had the intuitive prediction that segregating information by categories would encourage a focus on the target category. After all, integrating across categories in this display requires examining each of two or three options in the different categories and somehow integrating their utilities (perhaps weighted by the categories' probability, or perhaps not). This prediction was not borne out—in fact, the opposite occurred, as the category frame led to greater selection of the multiple-category choice, both when it was called for (Experiments 2A and 2B) and when it wasn't (Experiments 1, 2A, and 2B).

We find this effect puzzling, and our explanation for it must be tentative. One possibility is that presenting the effectiveness as percentages created a contrast effect, especially in the choice condition. Consider the choice frame in Table 4. When examining the Smith book, subjects would see that it was particularly appropriate for science majors, the most likely category: It was useful for 87% of science majors and 3% of business majors. This contrast may have made subjects think, “This is a really good book for science majors,” and choose it quite often. That is, the usefulness of the Smith book for the science majors was enhanced because of its inappropriateness for business majors. Thus, if subjects were reasoning in this way, they would be attempting to find the best choice for only a single category. None of the other choices have such a striking contrast (indeed, the multiple-category choice, Jones, goes in the opposite direction and is slightly more useful for the less likely category). Of course, the striking contrast is irrelevant when the goal is to make the best overall decision. If Smith book is particularly inappropriate for business majors, it is unlikely to be the best choice overall.

In the category frame, this contrast is not evident. The 87 and 3% are separated in different categories. The contrast within the science majors doesn't lead to a strong prediction (87% for Smith vs. 66% for Jones), so people might examine information in the other category, leading to more integrative predictions.

One simple way to evaluate the different frames is to consider the percent of best choices statistic, in which we coded the “best choice” as the multiple-category choice in the high uncertainty case and the target-category choice in the low uncertainty case. As Table 6 reveals, the frames are not obviously different. In Experiments 2A and 2B, although the category frame increased multiple-category use when it was appropriate, it also increased it when it was inappropriate. As a result, despite large differences in the use of multiple categories between the frames, the overall percent of best choices were similar for the two frames. In Experiment 1, the pattern is slightly different. The category frame increased multiple-category use only when it was inappropriate, and the percent of multiple-category choices was almost identical when these choices were appropriate. Thus, the choice frame led to a marginally larger percentage of best responses overall. A fair summary of all these results is that neither frame stands out as being significantly better than the other.

#### Recommendations and future directions

Overall, the results of this study are consistent with the similar research on category-based induction. Many people do not seem aware of when they should and should not use multiple categories. However, the overall tendency of responding was different here than in much of our past work. There (e.g., Ross and Murphy, 1996; Murphy and Ross, 2010b), people generally focused on one category even when they shouldn't have. Here, they often use multiple categories, even when they shouldn't. We suspect that the difference has to do with the presentation of probabilities and utilities in a tabular format, which encourages people to attempt to use as much of the information as possible. A study of induction that used a similar format (although it required button presses to reveal the information) similarly found that people used multiple categories (Murphy and Ross, 1999, Experiment 6), about 59% of the time. However, that study did not contain a low uncertainty condition and therefore was unable to reveal that such choices might be made even when they were not appropriate. These high levels of multiple-category use are generally not found with pictorial displays of categories or narrative presentation of common categories used in many studies of category-based induction.

One might question, then, whether such displays are helpful or harmful in providing people with necessary information for making decisions. Our results suggest that they could be helpful to the degree that they have been filtered for the individual to reflect possibilities and options that apply to him or her in particular. If they contain information that should be ignored or weighed less, people may not be very good at identifying and ignoring it. Furthermore, as revealed by the results of Experiment 2, even if they correctly identify some information as relevant only to unlikely outcomes, it might influence their decisions nonetheless. As the addition of highly unlikely alternatives to a set of possibilities has been found to change judged likelihood of likely possibilities in a nonnormative manner (Windschitl and Chambers, 2004), it seems that a general rule of thumb may be to omit information from very unlikely categories.

In thinking about how to tailor information, it is important to think about how costly errors are. Our best choice analysis revealed two different types of error: failure to take into account a relevant category by using a single category strategy in the high uncertainty condition, and failure to ignore an irrelevant category by using a multiple category strategy in the low uncertainty condition. The question of how best to present category information depends on whether it is more costly to ignore a relevant category or to consider an irrelevant alternative. Of course, which of these errors is worse depends on the costs and benefits associated with each choice, an issue not addressed by our stimuli (as we used novel categories and choices so that subjects would assume the costs and benefits of each choice as equal). However, one can argue that the use of multiple categories when only one is relevant is worse than using only one category when multiple ones are relevant. In the first case, one is relying on information that may very well be wrong (a category that is likely incorrect); in the second, one is relying on a subset of the accurate information available. Thus, it may be unwise to attempt to increase use of multiple options if that use is likely to be indiscriminate. Future research should investigate how subjects integrate information about choice costs and benefits into their decisions. This type of information may draw attention to outcomes and lead subjects to be more careful with their decisions. In fact, research on category-based induction under uncertainty has shown that an emphasis on the costs of ignoring relevant alternatives can lead to more normative use of category information (Hayes and Newell, 2009; Zhu and Murphy, 2013).

How the inability to ignore unlikely alternatives operates in situations where there are more categories or possibilities is an important question for future research. In our low uncertainty scenarios there were only two possible categories: one relevant (target category) and one irrelevant (alternative category). In our experiments, information from an irrelevant category was weighed too heavily while information from relevant categories was often not weighed heavily enough. In a situation where there are more possibilities, one could imagine there being several relevant and irrelevant categories. In such a situation, subjects may start to discard information from some categories because the more categories there are, the more challenging it is to consider or average over all possibilities. Indiscriminatingly considering presented information whether or not it's relevant, as our subjects did, could lead to irrelevant categories being considered at the expense of relevant ones. However, it is also possible that if more categories lead subjects to simplify their decisions, then they will do so selectively and discard information from less likely possibilities first. Future research will be necessary to discover how people behave under these more challenging conditions.

### Conflict of interest statement

The authors declare that the research was conducted in the absence of any commercial or financial relationships that could be construed as a potential conflict of interest.

## Footnotes

^1^This calculation assumes that the categories of rabbit and beetle are mutually exclusive and exhaustive.

^2^In the low uncertainty condition, the Smith book was 61.9% likely to be useful and the Jones book was 42.9% likely to be successful according to the probability calculation describes above. However, our suspicion is that people simply ignore the much less likely category.

^3^Unless the action involves a property known about cows but not known about horses. However, that was not the case for any of our items, as relevant property information was provided for every category.
