# Supplementary material for: Decision making under uncertain categorization
Source: Front Psychol. 2014 Sep 11;5:991. doi: 10.3389/fpsyg.2014.00991 (PMC4160964; doi:10.3389/fpsyg.2014.00991)
Supplement: Supplementary file 1 [file Table1.DOCX]

**Supplementary Material - Table S1**

Vignettes used in all experiments

| Text* | Experiment 1 | Experiments 2a and 2b |
| --- | --- | --- |
| Marjorie is packing her bag for the day and remembers that she has an appointment to talk with a student from her class about career options. She wants to bring her student a book that is relevant to her interests. The problem is that Marjorie only has room in her bag for one book and she can’t remember which of her students she is speaking with today, because her calendar was erased in a computer malfunction. Her class is made up of 65% (95%) science majors and 35% (5%) business majors. The types of jobs they are looking for are very different. The three books (one by Jones, one by Kendall, and one by Smith) about careers for recent college graduates that Marjorie has focus on different topics and are differentially useful for the different majors. Please press the next button to answer a question before you see this information and help Marjorie make a decision about what book to bring. | Science Majors (more likely)  Jones book is useful for 41% of science majors  Smith book is useful for 65% of science majors  Business Majors (less likely)  Jones book is useful for 78% of business majors  Smith book is useful for 3% of business majors | Science Majors (more likely)  87% find Smith book useful  1% find Kendall book useful  66% find Jones book useful  Business Majors (less likely)  3% find Smith book useful  96% find Kendall book useful  86% find Jones book useful |
| Brian visited the dermatologist because he had a mysterious rash. After carefully examining the rash, the doctor said, “I’m not completely sure what you have. This rash could only be caused by one of two conditions, daxitis or wuglosis. Most rashes like yours, about 62% (92%), are caused by daxitis, but about 38% (8%) are caused by wuglosis. There are three possible treatments: gamma blockers, alphaTr inhibitors, and ion promoters. The effectiveness of the treatments depends on which condition you have.” She then presented the following information about the possible conditions and treatments. Please press the next button to answer a question before you see this information and help Brian make a decision about his treatment. | Daxitis (more likely)  Gamma Blocker success rate is 36% for daxitis  Alpha-Tr Inhibitor success rate is 60% for daxitis  Wuglosis (less likely)  Gamma Blocker success rate is 73% for wuglosis  Alpha-Tr Inhibitor success rate is 1% for wuglosis | Daxitis (more likely)  Ion Promoter success rate is 1%  Alpha-Tr Inhibitior success rate is 63%  Gamma Blocker success rate is 88%  Wuglosis (less likely)  Ion Promoter success rate is 94%  Alpha-Tr Inhibitior success rate is 85%  Gamma Blocker success rate is 2% |
| Ben saw a mouse run under the radiator in his apartment. Being very squeamish and thus determined to rid his apartment of the pest, he consulted an exterminator to see how best to get rid of it. Upon examining the evidence of its existence (droppings, bite marks, etc.), the exterminator said, “Based on what I’ve seen I think it’s about 60% (90%) likely it’s a deer mouse, and there’s maybe a 40% (10%) chance it’s a white-footed mouse. There are three chemicals we could use to get rid of them: Miocide, Lerocide and Carbocide. The effectiveness of the chemicals depends on which type of mouse you have.” He then presented the following information about the possible rodents and solutions.  Please press the next button to answer a question before you see this information and help Ben make a decision about which chemical to use. | Deer Mice (more likely)  Miocide success rate is 62% for deer mice  Carbocide success rate is 38% for deer mice  White-Footed Mice (less likely)  Miocide success rate is 3% for white-footed mice  Carbocide success rate is 75% for white-footed mice | Deer Mice (more likely)  Lerocide success rate is 2%  Carbocide success rate is 65%  Miocide success rate is 90%  White-Footed Mice (less likely)  Lerocide success rate is 92%  Carbocide success rate is 79%  Miocide success rate is 3% |
| Ken is a personal assistant. His current task is to buy bug repellent for his boss’s trip to South America. At the store, Ken asks the sales associate what type of bug repellant he should buy. The sales associate says, “Well, it depends on whether your boss is going to be the rainforest or in a city. We carry three types of bug repellent: spinosad spray, calloric spray and allosteric spray. The effectiveness of these types of bug repellent differs depending on the environment.” He then gives Ken a brochure with the information below. Ken doesn’t know whether his boss is going to the city or the rainforest. His thinks that there’s a 60% (90%) chance that his boss is going to the rainforest and a 40% (10%) chance that he is going to the city. Ken knows that his boss specifically told him where he was going to be so he could buy the most effective bug spray, but he can’t remember the destination now, because he was supposed to buy the repellant last week and forgot to do it. He only has enough money with him to buy one product, and his boss’s car to the airport comes in 10 minutes.  Please press the next button to answer a question before you see the information about bug repellent effectiveness presented in the brochure and help Ken make a decision about which product to buy. | Rainforest (more likely)  Effectiveness of allosteric spray is 61% for rainforests  Effectiveness of spinosaid spray is 37% for rainforests  City (less likely)  Effectiveness of allosteric spray is 2% for cities  Effectiveness of spinosaid spray is 74% for cities | Rainforest (more likely)  Spinosaid spray is 64% effective  Allosteric spray is 92% effective  Calloric spray is 2% effective  City (less likely)  Spinosaid spray is 88% effective  Allosteric spray is 4% effective  Calloric spray is 95% effective |
| Jen was trying to get a medical residency. As you may know, medical students apply for a number of residencies, and a computer program matches them with one of them. Given her background, Jen had a decent chance of getting a residency in either dermatology or oncology, so she applied for an equal number of each. (Of course, she can only be assigned to one residency.) Her adviser told her that she had about a 65% (95%) chance of getting a dermatology residency and a 35% (5%) chance of getting an oncology residency. While she is waiting to hear her fate, Jen can take one of three medical technology courses, one in the use of lasers, one in the use of wireless monitoring devices, or one in the use of robotic surgery. She has ascertained that the classes would be differentially useful for different kinds of residencies, depending on exactly what hospital she went to and what programs they have there. Please press the next button to answer a question before you see this information and help Jen make a decision about which class to take. | Dermatology (more likely)  Laser class will be useful for 40% of Dermatology programs  Robotics class will be useful for 64% of Dermatology programs  Oncology (less likely)  Laser class will be useful for 77% of Oncology programs  Robotics class will be useful for 5% of Oncology programs | Dermatology (more likely)  Laser class useful for 64% of programs  Robotics class useful for 85% of programs  Wireless monitoring device class useful for 2% of programs  Oncology (less likely)  Laser class useful for 86% of programs  Robotics class useful for 4% of programs  Wireless monitoring device class useful for 96% of programs |
| Samantha is preparing to go on a long vacation. She is unsure where she is going to stay during the last week of her vacation in Spain, because her friend who is already there is going to find a youth hostel or a hotel for them. About 60% (90%) of the accommodations in her budget are youth hostels and about 40% (10%) are hotels. There are three competing travel organizations that provide discounts on these accommodations to its members: Travel Club, Inc., World Wide Adventures Club, and Backpackers International. As she only wants to pay dues for one organization, Samantha is trying to decide which to join. She knows that the two types of accommodation vary in how likely they are to offer discounts to members of each organization. This information is below. Please press the next button to answer a question before you see this information and help Samantha make a decision about which organization to join. | Youth Hostels (more likely)  Discounts for Backpackers International members at 63% of youth hostels  Discounts for Travel Club, Inc. members at 39% of youth hostels  Hotel (less likely)  Discounts for Backpackers International members at 4% of hotels  Discounts for Travel Club, Inc. members at 76% of hotels | Youth Hostels (more likely)  90% offer discounts to Travel Club, Inc. members  3% offer discounts to World Wide Adventures Club members  65% offer discounts to Backpackers International members  Hotel (less likely)  5% offer discounts to Travel Club, Inc. members  93% offer discounts to World Wide Adventures Club members  80% offer discounts to Backpackers International members |

* Percentages outside of parentheses were used in the high uncertainty condition. Percentages in parentheses were used in the low uncertainty condition.
